# Assessment of the Volatile Profiles and Identification of Differentiating Aromas of Wild Undervalued Plants

**DOI:** 10.3389/fnut.2022.912680

**Published:** 2022-07-08

**Authors:** Tamara Fukalova Fukalova, Estela Moreno-Peris, María Dolores García-Martínez, María Dolores Raigón Jiménez

**Affiliations:** ^1^Facultad de Ciencias Químicas, Laboratorio de Fitoquímica y Productos Biológicos, Universidad Central del Ecuador, Quito, Ecuador; ^2^Instituto de Conservación y Mejora de la Agrobiodiversidad Valenciana, Universitat Politècnica de València, Valencia, Spain

**Keywords:** volatile profiles, differentiating aroma, undervalued species, organoleptic qualities, seasonality

## Abstract

Wild edible plants have played an important role in traditional diets, including the Mediterranean diet. Many of these plants have acquired an undervalued status, since they are under-appreciated in terms of their nutritional, organoleptic qualities, or their seasonality. However, some of these species are still used in local gastronomy for their aromatic and taste characteristics. This study has investigated the quantitative and qualitative aromatic characteristics of seven undervalued wild plants that determine their organoleptic characteristics. Volatiles of the fresh leaves of each species have been determined by head-space solid-phase microextraction, a sensitive and solvent-free technique, coupled with gas chromatography and mass spectrometry. A total of 37 compounds with remarkable quantitative and qualitative differences were identified. In general, benzenoids and monoterpenoids were the most abundant groups, while branched unsaturated hydrocarbons, fatty alcohols, and sesquiterpenoids were the minor groups. Benzyl nitrile, benzyl isothiocyanate, p-cymene, and 2-hexenal were the main individual volatiles, while benzyl alcohol, eugenol, and α-copaene were the differentiating aromas. The results display that the undervalued species studied could be a suitable choice to include as new environmentally friendly crops, providing a double benefit to producers, because they are a possible way to achieve sustainable production systems, and they are an alternative for consumers, because these plants provide flavors that have high organoleptic qualities.

## Introduction

Many wild edible plants are characteristic of their seasonality, and local populations value these plants for their organoleptic properties. Traditionally, in the Mediterranean area, wild plants are used as food sources and condiments in the preparation of local dishes, for their aroma and flavor imparted by volatile constituents. They are well adapted to local growth conditions due to their resistance to climatic changes, which can be useful for alternative sustainable crops that bring new flavors and aromas. Furthermore, wild plants are very rich in fiber, minerals, vitamins, polyphenols, and antioxidants (1–3). Several studies confirm the nutritional and functional quality of wild plants, which is why they are appreciated in regional cuisines (4–6). The ways of using the plants depend on local traditions: They can be used fresh (salads) or cooked (soups and stews); alone or in combination with other species (7–10).

These diverse Mediterranean food traditions are part of the intangible cultural heritage that must be preserved in the face of modern lifestyles and a poorly differentiated diet (11, 12). Safeguarding traditional culinary knowledge could help reduce food poverty in the current climate emergency, promoting food security and increasing the value of local resources, as well as diversifying diets that are beneficial for health. When used fresh, the most useful parts of the plant are the leaves and flowers, which stand out because of their organoleptic characteristics. These characteristics include the visual appearance, the textures, and the aromatic profile, the main sensory attributes by which wild edible plants are valued. In general, the taste and smell continue to be powerful determinants of food selection, and consumers rank these elements as their main reason for choosing a food (13). The principal constituents of the sensory profiles are volatile compounds that enhance aroma and flavor, having a considerable impact as quality parameters and consumer preferences (14). Among the factors that influence the concentration of these volatile organic compounds (VOCs) are the genetic diversity of species and environmental conditions (15). All plants emit volatile compounds that are species-specific and are involved in certain ecological interaction, providing adaptative characteristics under strong environmental selection (14, 16, 17). Volatile compounds are synthesized mainly by chemical or enzymatic pathways, accumulating in plant organs such as leaves (18), flowers (19), or fruit (20). Currently, more than 1,700 volatile compounds have been identified from more than 90 plant families, constituting approximately 1% of all known plant secondary metabolites (21, 22). Each volatile compound is characterized by an odor threshold, so even if the qualitative composition of different samples is almost the same, the aroma may vary when relative proportions are dissimilar (23).

The differentiating aroma of each plant makes them attractive for harvesting and depends on the season. The wide varieties of volatile aromatic constituents are usually studied with principal attention given to antimicrobial and antioxidant activity, among other things (22, 24–28). Besides this, the review article by Goto et al. (29) highlights that the consumption of several wild plants also provides improvements in certain conditions, such as diabetes mellitus, hyperlipidemia, and cardiovascular diseases, because they contain many bioactive phytochemicals, especially terpenoids. These phytochemicals constitute some of the largest families of natural products that have a beneficial effect on health. Therefore, the relationship between diet and health could be considered a fundamental factor for healthy nutrition and well-being, while the aromatic profile could act as a stimulus for this synergy (13, 30). In addition, supported by broad health benefits, interest in this area continues to help to identify undervalued species as potential crop sources, as they may represent an underexploited source of new sustainable crops (31, 32).

Despite extensive culinary uses, there is little information on the aroma compounds of wild edible plants that add organoleptic quality to food sources. Determination of these volatile compounds from wild edible species has acquired momentum in recent years, mainly due to their bioactivities that remain intact when consumed fresh. The identification of certain constituents could make it possible to establish the qualitative indicators of the organoleptic characteristics and the optimal state of harvest of the undervalued species. According to (33), these selected plants have cultural relevance and are deeply rooted in the traditional cuisine of the Valencian coast. As far as we know, there are few works describing these characteristics, and we have not found any report analyzing the volatile profiles of the wild edible plants described in this study. For this reason, the focus has been on highlighting the organoleptic value of these species. More specifically, the aim of this study was to evaluate and compare the aromatic profiles of seven undervalued edible plants that form part of biocultural diversity and regional culinary traditions (34). To meet this global objective, the research process was (a) to select the undervalued edible plant species potentially used for consumption in the study area according to seasonality; (b) to identify the organoleptic matrix, such as the volatile profiles, to find the differentiating aroma and flavor characteristics of each species; and (c) to recognize qualitative–quantitative differences and similarities between the volatile profiles. The plants selected in the study were *Portulaca oleracea* L. and *Porophyllum ruderale* (Jacq.) Cass., which relate to the spring–summer season; *Stellaria media* (L.) Vill, *Tropaeolum majus* L., *Sonchus oleraceus* L., *Chenopodium album* L., and *Diplotaxis erucoides* (L.) DC relate to the autumn–winter season. Their volatile profiles were evaluated by the combined techniques of solid-phase microextraction (SPME) and integrated gas chromatography by a mass spectrophotometer (GC-MS). This technique was chosen for not involving temperatures that affect the stability of volatile compounds and allowing the unalterable metabolites of the aromatic profiles to be known.

The identification of the volatiles in the seven undervalued plants is intended to be a precedent to assist advances in the inclusion of these plants in cuisines, as an alternative to conventional vegetables, to diversify the intake of a balanced and healthy diet. At the same time, identification of the volatile compounds could provide a better understanding of the organoleptic effect on the particular flavor of each species with a wide presence in the corresponding season and promote traditional gastronomy. This study may also guide the future commercial exploitation of these species, not only as alternative food sources and crops in agronomic innovations, but also as an aid in making informed decisions on the collection and standardization of quality parameters.

## Materials and Methods

### Plant Material

**Figure 1:**
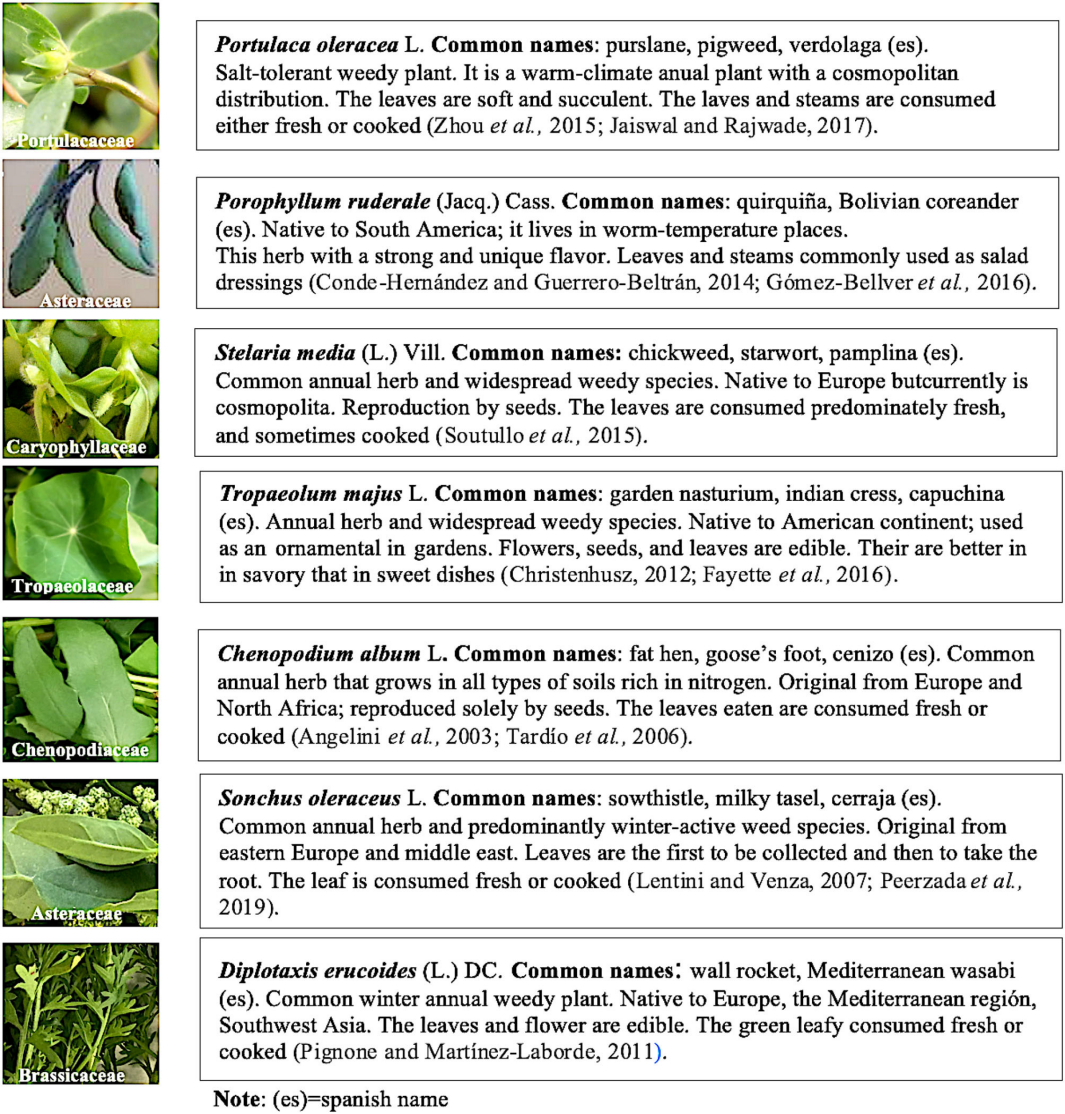
Botanical families and a brief description of the seven wild edible species selected for study.

The seven edible undervalued plants were selected as plant material from different botanical families. The species corresponded to the wild population and were collected during two growing seasons in 2020. The replicates harvested were collected on the same day, expanding the geographic diversity but not the temporal one, so as not to include new variables in the study. The aerial parts of fresh samples were visually inspected before sampling, and only intact and healthy plants were collected, with the assistance of the Ecological Cooperative in the rural environment areas of the coast of the province of Valencia (Spain). The area is located within 39°45'13” North and 0°12′21″West, with SCI code ES0000148 (35). A brief description of each plant is summarized in Figure 1.

The undervalued species with abundance in their respective season appear spontaneously and present as weeds. Samples of these fresh plants were manually cleaned to remove soil particles, and whole leaves were selected for analysis.

### Sample Preparation

The extraction of volatile compounds without the addition of solvents has been successfully used in plant materials. Groups of plant material (leaves) of each species were collected, chopped, and stored at −80°C prior to gas chromatography–mass spectrometry as independent biological replicates. About 1.5 mg of each leaf sample was introduced into a hermetically sealed 22 mL vial, and it was incubated at 40°C for 30 min.

### Extraction of Volatiles

Isolation of volatile constituents was performed by a head-space solid-phase microextraction (HS/SPME) technique. SPME Fiber Assembly, 65 μm PDMS/DVB Stableflex (Supelco, Bellefonte, PA, USA), was used to collect and concentrate the aroma compounds. Before the samples were loaded, the fiber was inserted into a GC injector (270°C) and held for 1 h, according to (36). After a 40 min equilibration period at 40°C, the fiber was inserted into the incubated vial splitless mode at 250°C for 30 s in the gas chromatograph injection port.

### Volatile Profile Analysis

The analysis of volatiles was performed by 6890 N gas chromatography and mass spectrometry (GC-MS), networked to a 59,73 Inert Mass Selective Detector (Agilent Technologies; Santa Clara, CA, USA). The analytical conditions were as follows: stationary phase HP-5MS J&W silica capillary column (30 m × 0.25 mm i.d. x 0.25 μm thickness film; 5%-phenyl-95% methylpolysiloxane); helium carried gas at a constant flow of 1 mL·min^−1^; transfer line was maintained at 220°C. Initial temperature (40°C) was maintained for 1 min, Ramp 1 from 5°C min^−1^ up to 200°C for 1 min^−1^, and Ramp 2 from 15°C min^−1^ up to 250°C for 3 min. The electron impact mode with ionization energy of 70 eV (source temperature 225°C) was used for detection by the mass spectrometer, and the acquisition was performed in scan mode (mass range *m/z* 35–350 amu). MSD ChemStation E.02.02.1431 (Agilent Technologies) was used to perform chromatograms and mass spectra. Fiber cleaning between samples was 5 min at 250°C.

### Determination of Volatiles

The identification of volatiles took place by combining two different aspects: a comparison of their mass spectra and GC retention time with commercial standards (RS, Sigma-Aldrich Co, Taufkirchen, Germany) and the matching degree in the NIST 2017 Mass Spectral library. Finally, for quantification, a total ion current chromatogram (TIC) was employed to integrate the peak area of each compound, similar to other previous works (37, 38). In this work, the compounds considered as identified are those whose mass spectrum shows a height matching the NIST library (>80%), or according to the available standards.

### Statistical Analysis

Three replicates were used to obtain the mean values for the levels of volatile compounds and the content of each one. Relative abundances for individual compounds were also calculated as the ratio between the GC-peak area against the added area of total volatiles identified and expressed as percentages. The dependent variable defined the volatiles, and the independent variable was the species. The dataset was subjected to one-way analysis of variance (ANOVA). Significant differences were assessed by multiple comparisons of means (Tukey's contrast, with a confidence limit based on 95%, *p* < 0.05). The Statgraphics Plus version 5.1 (Manugistics Inc., Rockville, MD, USA) was used for statistics.

In addition, the dataset was also subjected to principal component analysis (PCA) and hierarchical cluster analysis by means of a web ClustVis tool developed with the R package version 0.10.2.1 for R statistic software, to evaluate the similarity and variation between samples.

## Results

### Volatile Compounds Identified

The samples showed significant differences in their total levels of volatiles identified, and therefore, there was a considerable diversity in this trait. The analysis of volatiles yielded thirty-seven identified compounds, which were grouped into 11 chemical families, based on their structural characteristics. Table 1 details the mean value of the GC-peak area of each volatile; it also includes the individual code and the identification method, the retention time, and the Kovats index, as well as the *p*-value (cutoff significance of *p* < 0.05).

**Table 1:** Mean values (GC-peak area unit x 10^6^) for individual volatile compound identified in the leaves of the seven undervalued species and levels of significance (*p*-value).

					**Peak area**							
**N**°****	**Compounds name**	**Id**	**RT**	**RI**	***T. majus***	***S. media***	***S. oleraceus***	***C. album***	***D. erucoides***	***P. oleracea***	***P. ruderale***	***p*-value**
1	dimethyl sulfide	MS	1.785	520	–	–	1.347a	–	–	–	0.166b	0.0061
2	1-butanol, 3-methyl	MS	3.501	736	–	–	1.039	0.183	–	0.677	–	0.4226
3	hexanal	RF	4.95	800	0.128d	13.975a	0.590c	0.718c	0.236d	0.054d	5.8b	0.0079
4	2-hexenal-E	MS	5.765	851	–	0.603	0.152	0.292	0.088	–	0.341	0.1252
5	2-hexenal	RF	5.923	854	0.367c	22.664a	6.558b	7.993b	3.142c	–	14.216b	0.0185
6	trans-2-hexenol	MS	6.285	862	–	4.564a	2.515b	0.149c	0.471c	–	–	0.0026
7	1-hexanol	RF	6.335	868	0.060d	13.893a	0.925c	4.015b	0.186c	0.158c	–	0.0019
8	2,4-hexadienal-(E,E)	MS	7.433	911	–	1.053b	0.242c	0.603c	–	–	3.876a	0.0098
9	benzaldehyde	MS	8.828	962	122.878a	4.179c	6.2c	4.914c	65.748b	0.588c	1.08c	0.0000
10	6-methyl-5-hepten-2-one	MS	9.636	986	0.070b	–	0.129b	0.204a	–	–	–	0.0234
11	β-myrcene	MS	9.754	991	0.066c	–	0.321b	–	–	–	37.531a	0.0218
12	2,4-heptadienal-(E,E)	MS	10.301	1012	1.479	12.612	3191,000	3.203	0.568	–	1,936	0.0659
13	α-terpinene	MS	10.503	1017	–	–	–	0.363b	–	–	45.746a	0.0140
14	p-cymene	MS	10.701	1025	–	0.196b	0.167b	0.176b	–	0.136b	98.929a	0.0018
15	d-limonene	RF	10.851	1030	0.024c	0.100c	0.260b	0.487a	–	0.156c	–	0.0018
16	1,8-cineole (eucalyptol)	RF	10.934	1032	0.052d	0.221b	–	0.137c	–	0.333b	0.458a	0.0052
17	benzyl alcohol	MS	11.123	1036	0.640	–	–	–	–	–	–	–
18	(E)-β-ocimene	MS	11.137	1049	–	–	–	–	–	–	3,88E+03	–
19	benzeneacetaldehyde	MS	11.302	1045	0.380d	0.554c	11.747a	1.420c	–	0.295c	2.451b	0.0000
20	(Z)-β-ocimene	MS	11.444	1038	0.105b	0.042c	0.267b	0.284b	–	0.079c	55.402a	0.0035
21	y-terpinene	MS	11.775	1060	–	–	0.086b	0.052b	–	–	77.857a	0.0011
22	3,5-octadien-2-one, (E,E)	MS	12.131	1073	–	8.121	0.251	0.378	–	–	0.373	0.0853
23	2-isopropyl-3methoxypyrazine	MS	12.835	1097	–	–	–	–	–	0.121	–	–
24	linalool	RF	12.992	1099	–	–	–	0.102b	–	1.178a	–	0.0093
25	nonanal	RF	13.109	1104	0.689b	0.505b	0.102b	0.079c	0.041c	–	0.941a	0.0337
26	benzyl isocyanate	MS	13.658	1131	35.730a	–	–	–	0.463b	–	–	0.0053
27	benzyl nitrile	MS	14.137	1144	19.302b	0.467c	0.212c	0.199c	1861.725a	–	0.482c	0.0000
28	2,6-nonadienal-(E,Z)	MS	14.547	1155	0.060	1.709	0.083	0.065	–	–	0.112	0.0794
29	2-sec-butyl-3-methoxypyrazine	RF	15.116	1175	–	0.127c	0.128c	–	3.839a	1.463b	–	0.0000
30	2-isobutyl-3-methoxypyrazine	MS	15.349	1183	0.208b	–	–	–	0.216b	0.327b	0.639a	0.0073
31	decanal	RF	16.071	1206	0.061c	0.280b	0.020c	0.019c	0.013c	–	15.115a	0.0012
32	β-cyclocitral	MS	16.515	1220	0.167b	3.470a	0.257b	0.282b	0.205b	0.072b	0.244b	0.0016
33	eugenol	MS	20.223	1357	–	–	–	–	–	–	0.743	–
34	benzy isothiocyanate	MS	20.435	1364	3507.83a	0.570c	0.826c	2.689c	55.277b	0.063c	0.806	0.0000
35	α-copaene	MS	20.757	1376	–	–	–	–	–	–	0.992	–
36	β-caryophyllene	RF	21.901	1419	0.229b	0.067c	0.052c	0.060c	0.031d	–	7.447a	0.0018
37	trans-β-ionone	MS	23.503	1486	0.499b	11.252a	0.598b	0.736b	1.278b	0.134b	1.436b	0.0004

*N°: consecutive number; Code, coding of each volatile components; Id, identification method (RF = reference commercial standard, MS = comparison of mass spectrum with NIST library); RT, Retention time (min); RI, Kovats Retention index; (a–d) letters indicate significant differences (p < 0.05)*.

The abundance of each chemical family and their respective components were calculated and reported in the peak area relative percentage (Table 2). In general, among eleven chemical families, benzenoids (I), monoterpenoids (II), and medium-chain aldehydes (III) were the majority, with eight volatile components in each of them. In the minority families, the lowest relative concentration was presented by pyrazines (IV), with three compounds, followed by fatty alcohols (VI), sesquiterpenoids (VIII), and organooxygen (IX), and with two compounds for each family. Only one volatile corresponded to the chemical families of alcohols (VII), unsaturated branched hydrocarbons (V), ketones (IX), and organosulfur compounds (X). Regarding the species, benzenoids were the dominant chemical families in *T. majus* (99.89%) and *D. erucoides* (99.48%). Monoterpenoids were abundant in *P. ruderale* (63.83%) and medium-chain aldehydes in *S. media* (50.77%).

**Table 2:** Mean relative abundances percentages (%) of chemical families and individual volatile compounds identified in the leaves of the seven undervalued species.

**Chemical family/Compounds name**		**Code**	***T. majus***	***S. media***	***S. oleraceus***	***C. album***	***D. erucoides***	***P. oleracea***	***P. ruderale***
**I. Benzenoids**		99.89	5.70	49.61	30.75	99.48	16.21	1.74
1	benzaldehyde	BZ	3.33	4.13	16.20	16.35	3.30	10.08	0.29
2	benzyl alcohol	BA	0.02	–	–	–	–	–	–
3	benzeneacetaldehyde	BC	0.01	0.55	30.70	4.72	–	5.06	0.65
4	benzyl isocyanate	BI	0.97	–	–	–	0.02	–	–
5	benzyl nitrile	BN	0.52	0.46	0.55	0.66	93.39	–	0.13
6	eugenol	EU	–	–	–	–	–	–	0.2
7	benzy isothiocyanate	BT	95.04	0.56	2.16	9.02	2.77	1.07	0.21
8	α-copaene	CO	–	–	–	–	–	–	0.26
**II. Monoterpenoids**		0.01	0.51	2.66	5.15	-	32.24	63.83
9	β-myrcene	MY	0.00	–	0.84	–	–	–	9.90
10	α-terpinene	AT	–	–	–	1.21	–	–	12.07
11	p-cymene	PC	–	0.19	0.44	0.58	–	2.33	26.10
12	d-limonene	LI	0.00	0.10	0.68	1.62	–	2.67	–
13	1,8-cineole (eucalyptol)	CI	0.00	0.22	–	0.46	–	5.70	0.12
14	(E)-β-ocimene	OC	–	–	–	–	–	–	1.02
15	(Z)-β-ocimene	OM	0.00	0.04	0.70	0.94	–	1.36	14.62
16	linalool	LN	–	–	–	0.34	–	20.18	–
**III. Medium-chain aldehydes**		0.07	50.77	28.59	43.42	0.20	0.93	11.17
17	hexanal	HA	0.00	13.81	1.54	2.41	0.01	0.93	1.53
18	2-hexenal-E	HE	–	0.60	0.40	0.98	0.00	–	0.09
19	2-hexenal	HD	0.01	22.39	17.14	26.82	0.16	–	3.75
20	2,4-hexadienal-(E,E)	HX	–	1.04	0.63	2.01	–	–	1.02
21	2,4-heptadienal-(E,E)	HP	0.04	12.46	8.34	10.66	0.03	–	0.51
22	nonanal	NN	0.02	0.50	0.27	0.26	0.00	–	0.25
23	2,6-nonadienal-(E,Z)	ND	0.00	1.69	0.22	0.22	–	–	0.03
24	decanal	DE	0.00	0.28	0.05	0.06	0.00	–	3.99
**IV. Pyrazines**		0.01	0.13	0.33	-	0.20	32.74	0.17
25	2-isopropyl-3methoxypyrazine	IP	–	–	–	–	–	2.07	–
26	2-sec-butyl-3-methoxypyrazine	SB	–	0.13	0.33	–	0.19	25.07	–
27	2-isobutyl-3-methoxypyrazine	IB	0.01	–	–	–	0.01	5.60	0.17
**V. B.unsaturate hydrocarbons**								
28	y-terpinene	TP	–	–	0.22	0.17	–	–	20.54
**VI. Fatty alcohols**		-	18.23	8.99	13.97	0.03	2.72	-
29	trans-2-hexenol	TH	–	4.51	6.57	0.50	0.02	–	–
30	1-hexanol	HL	0.00	13.72	2.42	13.47	0.01	2.72	–
**VII. Alcohols**		-						
31	1-butanol, 3-methyl	BU	–	–	2.72	0.61	–	11.60	–
**VIII. Sesquiterpenoids**		0.02	11.19	1.70	2.67	0.06	2.30	3.34
32	β-caryophyllene	CP	0.01	0.07	0.14	0.20	0.00	–	1.96
33	trans-β-ionone	IO	0.01	11.12	1.56	2.47	0.06	2.30	0.38
**IX. Ketones**		-						
34	3,5-octadien-2-one, (E,E)	OD	–	8.02	0.66	1.27	–	–	0.10
**X. Organosulfur compound**			-						
35	dimethyl sulfide	DS	–	–	3.52	–	–	–	0.04
**XI. Organooxigen compound**		-	3.43	1.01	1.63	0.01	1.23	0.06
36	6-methyl-5-hepten-2-one	MH	0.00	–	0.34	0.68	–	–	–
37	β-cyclocitral	CC	0.00	3.43	0.67	0.95	0.01	1.23	0.06

*Code, coding of each volatile components; majority families from I to III; minority families from IV to XI*.

**Figure 2:**
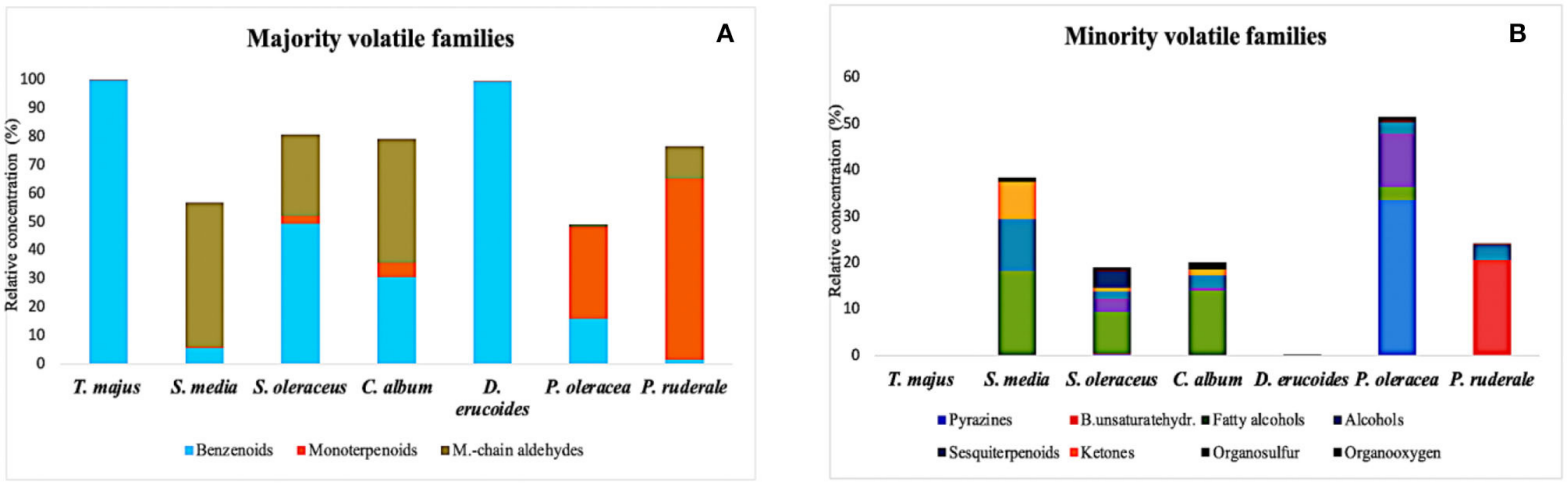
Chemical families of volatiles and their relative content in the leaves of the seven undervalued species. **(A)** majority volatile families; **(B)** minority volatile families.

The comparison of volatile compounds is presented in the leaves, and it showed that volatiles such as benzaldehyde (BZ), benzeneacetaldehyde (BC), benzyl isothiocyanate (BT), hexanal (HA), trans-β-ionone (IO), and β-cyclocitral (CC) were present in all species; some volatiles were present only in one species, being considered differentiating aromas. In *T. majus*, the differentiating aroma was benzyl alcohol (BA); in *P. ruderale*, it was eugenol (EU), α-copaene (CO), and (E)-β-ocimene (OC); in *P. oleracea*, it was 2-isopropyl-3-methoxypyrazine (IP); benzyl isocyanate (BI) was found only in the species *T. majus* and *D. erucoides*. The relative content of each chemical family, including the respective individual volatiles, is summarized in Figure 2.

#### Tropaeolum Majus L (Tropaeolaceae)

It was possible to identify a total of 22 volatile metabolites in this species, most of them belonging to the benzenoids chemical family, followed by medium-chain aldehydes (0.07%). The principal volatile constituent was benzyl isothiocyanate (BT, 95.04%). The differentiating aroma found only in this species was benzyl alcohol (BA, 0.02%).

#### Stellaria Media (L.) Vill (Caryophyllaceae)

Regarding this species, a total of 23 volatile compounds were identified. The main chemical families were medium-chain aldehydes, fatty alcohols (18.23%), and sesquiterpenoids (11.19%). Between the individual volatile compounds identified, the abundant ones were 2-hexenal (HX, 22.39%) and hexanal (HA, 13.81%) from medium-chain aldehydes, and 1-hexanol (HL, 13.72%) from the fatty alcohols family. Trans-β-ionone (IO, 11.12%) represented the sesquiterpenoids family.

#### Sonchus Oleraceus L. (Asteraceae)

A total of 27 volatile compounds were identified, being mainly characterized by the benzenoids family (49.61%), followed by the medium-chain aldehydes family (28.59%). The volatile compounds with the greatest influence on the volatile composition of this species are benzeneacetaldehyde (BC, 30.7%), 2-hexenal (HX, 17.14%), and benzaldehyde (BZ, 16.20%).

#### Chenopodium Album L. (Chenopodiaceae)

Of the 27 identified volatiles, the families of medium-chain aldehydes (43.42%) and benzenoids (30.75%) predominated, followed by the fatty alcohols family (13.97%). The most abundant compounds of the predominant families were 2-hexenal (HX, 26.82%) and 2.4-heptadienal-(E,E) (HP, 10.66%), followed by benzaldehyde (BZ, 16.35%), respectively, whereas 1-hexanol (HL; 13.47%) was from minority families.

#### Diplotaxis Erucoides (L.) DC (Brassicaceae)

The profile of this species was characterized by 17 individual volatiles found, essentially, from the benzenoids family. At an individual level, the most predominant compound was benzyl nitrile (93.39%). Between the individual volatile compounds from minority families, the presence of 2-hexenal (HX, 0.16%) was highlighted.

#### Portulaca Oleracea L. (Portulacaceae)

It was possible to identify a total of 16 volatile compounds in this species, of which 32.74% were pyrazines and 32.24% monoterpenoids families, followed by benzenoids (16.21%) and alcohols (11.60%) ones. At the individual level, the compounds with the highest content were 2-sec-butyl-3-methoxypyrazine (SB, 25.07%) and linalool (LN, 20.18%). The volatile from minority families with the greatest amount was 1-butanol, 3-methyl (BU, 11.60%). The differentiating aroma found only in this species was 2-isopropyl-3-methoxypyrazine (IP, 2.07%).

#### Porophyllum Ruderale (Jacq.) Cass (Asteraceae)

Regarding the characterization of the volatile profile of this species, 27 volatile metabolites were identified, mainly families of monoterpenoids and branches unsaturated hydrocarbons (20.54%). Its main individual volatile constituents were p-cymene (PC, 26.10%) and γ-terpinene (TP, 20.54%), respectively. The differentiating aromas found only in this species were (E)-β-ocimene (OC, 1.02%), α-copaene (CO, 0.26%), and eugenol (EU, 0.2%).

### General Analysis

The general ANOVA analysis recognized the statistically significant effects of the species (*p*-value) on the content of volatile compounds, which are indicated in Table 1, with the letters as a super index for each constituent.

Most of the individual volatile compounds had significant differences between species (*p* < 0.05), except medium-chain aldehydes (code HE, HP, and ND), monoterpenoids (PC), and ketones (OD). These general results indicate a different behavior in the synthesis of individual volatile compounds, depending on each species and the specific environmental conditions of the season.

### Principal Component Analysis

**Figure 3:**
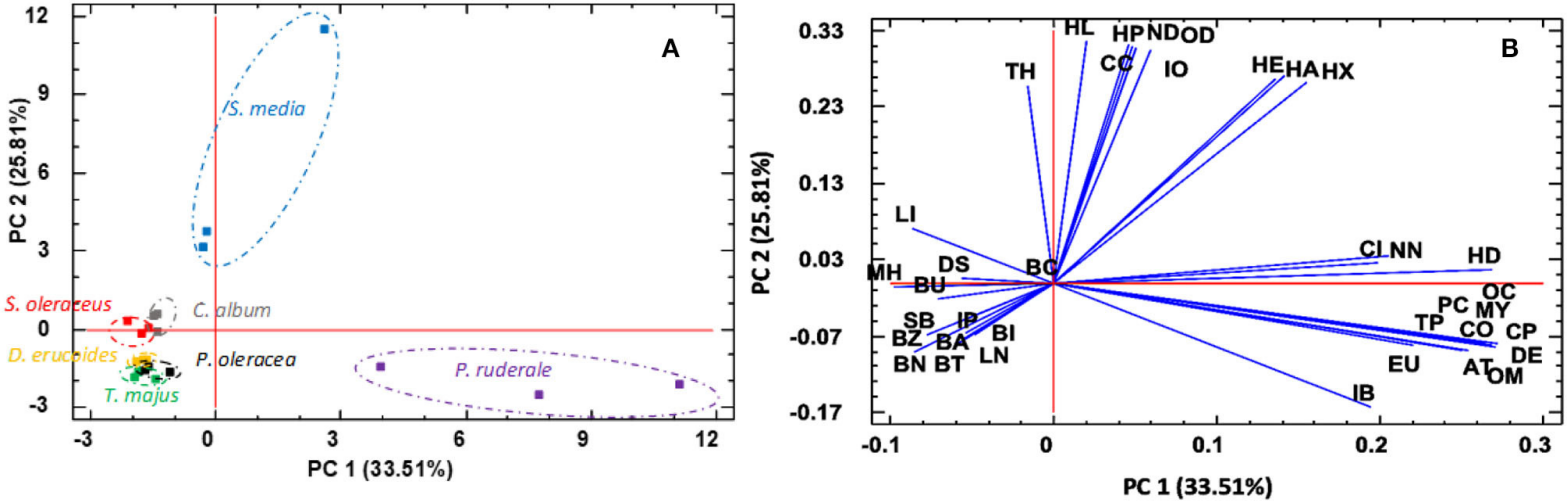
Principal component analysis (PCA) of the investigated samples. Scores **(A)** and loadings **(B)** of the two principal components on the matrix correlations were built using data from individual volatiles.

To reduce the dimensionality of the dataset containing many interrelated variables, the principal component analysis (PCA) method was applied, which allowed obtaining a reduced number of linear combinations of 37 identified aromatic components. In this case, six components have been extracted that explain 92.35% of the variability in the original data. Figure 3 shows the values of two principal components with greater strength, which explain more than 50% of the variability of the data, the first component (PC1) and second (PC2) accounted, respectively, 33.51% and 25.81% of the total variation. Figure 3A (the score) allows visualization of the sample distribution based on PC1 and PC2. For instance, three species, *D. erucoides, T. majus*, and *P. oleracea*, were spread along PC1 and PC2 at negative value for both. Two species *S. oleraceus* and *C. album* cluster around zero on PC2 and have been characterized by negative value of PC1. The species *P. ruderale* is characterized by high value of PC1 component and negative values of PC2. The species *S. media* was spread along PC2 at high positive value. Figure 3B (the loadings) shows the distribution of each of the identified volatile components has had in the classification of the species. The loading presentation indicated that the monoterpenoid and sesquiterpenoid families were predominantly important for species separation along PC1, while the medium-chain aldehyde family was predominantly important for species separation along PC2.

**Figure 4:**
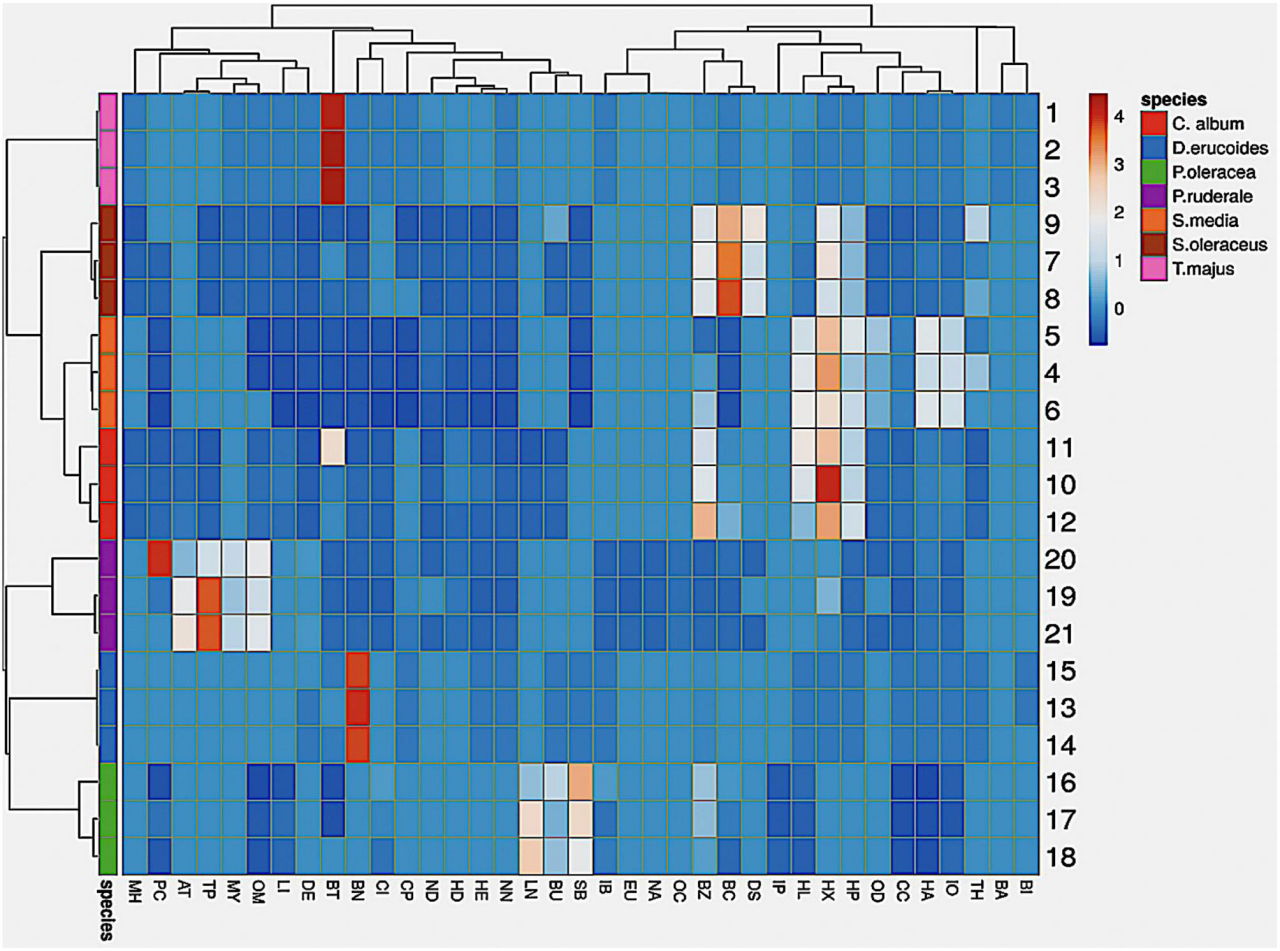
Hierarchical cluster analysis for identified volatile compounds in the seven undervalued species. Data represent the average value compounds (*n* = 3) for each studied plant. For the code of the compound identities, refer to Table 1.

Larger loadings indicate that the volatile has a strong effect on the corresponding PC. The most positive effects on the PC1 have compounds with the codes α-copaene (CO), β-myrcene (MY), α-terpinene (AT), β-caryophyllene (CP), (E)-β-ocimene (OC), (Z)-β-ocimene (OM), 2,4-hexadienal-(E,E) (HD), and decanal (DE), and the most negative effect the d-limonene, 5-methyl-5-hepten-2-one. The most positive effects on the PC2 have compounds with the codings hexanal (HA), 2-hexenal (HX), 2-hexenal (HE), 2,4-heptadienal-(E,E) (HP), 2,6-non-adienal-(E,Z) (ND), β-cyclocitral (CC), trans-β-ionone (IO), 3,5-octadien-2-one, (E,E) (OD), and the most negative effects α-terpinene and eugenol. Therefore, the between-species difference revealed by the preliminary PCA of volatile profiles is probably due to genotype diversity, leading to a different metabolic pattern rather than geoclimatic conditions. In addition, the relationship between individual compounds was also evaluated by the hierarchical cluster analysis (Figure 4).

This analysis grouped samples into two main clusters, where five subgroups are clearly differentiated. *S. oleraceus, S. media*, and *C. album* were the most numerous subgroup. These species showed a more homogeneous distribution of the chemical families and their respective compounds. A homogeneous presence was observed for the majority chemical families such as medium-chain aldehydes, followed by the benzenoids with less homogeneity, while the minorities were such families as fatty acids, ketones, and organooxygen compounds. Other subgroups contained a single species, which were *T. majus, P. ruderale, D. erucoides*, and *P. oleracea*, respectively. Each of these species was classified as an independent group due to the heterogeneity or absence of some volatile compounds and, above all, due to the presence of differentiating aromas. However, *T. majus* is grouped with the most numerous subgroup, while *D. erucoides* and *P. oleracea* could also be grouped in another subcluster. The only species that has a differential aromatic behavior is *P. ruderale*.

## Discussion

Edible wild plants are traditionally used for their flavor and aroma in food, to improve the attractiveness of everyday dishes and, probably, to prevent certain human diseases. Aroma-active volatile compounds of plants are found in very small quantities. Despite their small amounts, they can act as olfactory and taste stimuli. The wild edible species of this study belonged to different seasons of Mediterranean conditions, which were spring–summer and autumn–winter.

The contents of volatile compounds varied markedly among the seven undervalued species, each differing in their aroma profile. The typical aroma of vegetables depends on the number of volatiles, their chemical nature, and the synergic effects. Also, the portion of glycosidically bound volatiles is usually greater than that of free volatiles, making them an important potential source of flavor compounds (39). The relative amounts of these compounds and their odor threshold may be related to the time of harvest and habitat conditions. A total of 37 volatiles were found among seven species studied, and they were grouped into 11 chemical families, due to their qualitative and quantitative differences. All obtained profiles were shown to have a variety of proportions of identified volatile compounds. The most abundant groups according to GC-peak area units were benzenoids, monoterpenoids, and medium-chain aldehydes, whereas branched unsaturated hydrocarbons and fatty alcohols were minor groups, among others. 37 highlight that the volatile compounds synthesized by the botanical families to which the studied species belong are composed of twelve more common chemical families, among which are C5-branched chain compounds; nitrogen-containing compounds; aliphatic compounds; benzenoids and phenyl propanoids; sesquiterpenes; and irregular terpenes and monoterpenes, all of which were found in our study. At the individual level, a wide range of qualitative variability was also detected, with compounds such as benzaldehyde (BZ), benzyl isothiocyanate (BT), hexanal (HA), trans-ß-ionone (IO), and ß-cyclocitral (CC). A few compounds were only found in some species and were considered as differentiating aromas. Consequently, the differences of chemical groups were also very diverse at both quantitative and qualitative levels.

There is little evidence in the literature for volatile profiles from the fresh leaves of *T. majus*, since the flower is one of the most popular and best-known edible parts. Thus, study of the essential oil obtained by hydrodistillation of *T. majus* aerial parts (leaves and flowers), carried out by Lim (40), reported the content of eight carotenoids, fatty acids, flavonoids, and antibiotic tromalytes and found that the leaves were the primary site of benzylglucosinolate synthesis, with their contents increasing in the adult plant. On the contrary, (41) have reported high levels of glucosinolate glucotropaeolin concentration (between 48 to 78 μmol/g dry weight) in the dried leaves of *T. majus*, determined by HPLC analysis. After enzymatic activity, this glucosinolate transforms into benzyl isothiocyanate, a volatile compound first found in our research to be in abundance in this plant. In the study of the same authors, it is pointed out that similar enzymatic activity occurs when Tropaeolum leaves are freshly consumed, so the derived compound is absorbed in the intestine and excreted in the urine, exhibiting its antimicrobial activity (41). However, (42) have completed the characterization of the volatile compounds of Indian cress (*T. majus*) by GC-olfactometry/VIDEO-Sniff and HS-SPME-GCxGC-TOFMS, where they identified 44 odorant compounds and highlighted powerful sulfury and fruity notes. In our study, for the same species, organosulfur compounds were absent and benzyl isothiocyanate was classified as the benzenoids family (although with the presence of sulfur); this was substantially predominant, followed by benzaldehyde. The benzaldehyde compound has aromatic descriptors such as almond notes, roasted, and bitter (43, 44), while benzylglucosinolate accumulates in the mature plants of *Tropaeolum majus* L., increasing its content progressively from seeds until the leaves become the main site for its synthesis (45). The results of our study suggest that benzaldehyde and benzyl isothiocyanate in *T. majus* might be of special importance for the aroma of this one. Other secondary metabolites with strong anti-inflammatory activity and an important anti-infectious/antimicrobial action provided by aromatic esters and alcohols that were highlighted have also been described in other works (46). The volatile compound 1-hexanol (fatty alcohols family) was found in this study. Despite its scarcity, it is associated with a pungent aroma (47). A study by Kawada (48) has reported that pungent ingredients have anti-weight-gain properties and are being utilized for the development of functional foods. Both compounds, benzyl alcohol and benzyl isocyanate, identified in our study in *T. majus*, may be postulated as differentiating aromas, since the first is characteristic only of this species, and the other, due to its significant concentration, is compared to the other components from their volatile profile. The review article by (49) corroborates that benzyl alcohol has been reported to occur in nature, with the highest amounts observed in allium plants species. However, future studies are necessary to determine the differentiating aroma of this compound in the botanical family Tropaeolaceae (*T. majus*). In addition, according to (50), alcohols, aldehydes, and acetates are widely distributed in fresh green plant tissues and considered to be responsible for typical “green leaf” odor. On the contrary, the isothiocyanate identified has aromatic descriptors that characterize it as a “cabbage” aroma (51) and confer several bitter taste characteristics and black mustard-like notes, as well as giving beneficial bioactive effects (52).

Other species of our study (*D. erucoides*) also had a presence of benzyl isothiocyanate, although in much lower concentration. Its presence may be since both plants are from the Brassicales order, which is characterized by accumulate glucosinolate metabolites, which enzymatically hydrolyze into other volatile compounds by plant tissue damage (53). The derived products from hydrolysis of glucosinolates depend on the plant species and conditions in which the hydrolysis occurs. These hydrolysis products do not reach a toxic level for humans when they are included in a diet (54, 55). In *D. erucoides*, this hydrolysis can lead to considerable levels of isothiocyanates and nitriles (56). In addition, the intake of these compounds provides health benefits such as the reduction of the risk of cancer (57). In our case, the benzyl nitrile was the majority component from the benzenoid chemical family in *D. erucoides*. The nitrile component is also contributing toward pungency notes and several bitter tastes, in addition to providing the sulfurous aromatic attributes (53). This aromatic combination is modified by the presence of the other volatile components that affect the acceptance of the organoleptic characteristics of the plants (58). In particular, the volatile profiles of Valencian *D. erucoides* in baby-leaves stages grown in a substrate under controlled conditions in the study carried out by (59) were different, detecting only nine compounds vs. fifteen in this study, of which only one has coincided in our study (benzyl isothiocyanate). Likewise, the absence of the monoterpenoids family was observed. This difference suggests the importance of the state of maturity on the formation of the volatility profile and the optimal harvest time of the plants (60, 61). No differentiating aroma was detected in this species.

*P. ruderale* was the species where the greatest number of differentiating aromas was found. These important plant constituents were (E)-β-ocimene > α-copaene and eugenol. The research carried out by 61 described the flavor/aroma of (E)-β-ocimene as a “pleasant, warm herbaceous note” and noted that its presence depends on seasonal variation. In addition, this component is among the main volatile ones in parsley (*P. crispum* (Mill.) Nyx. Ex A.W. Hill) and peppermint (*Mentha x piperita* L.) (62). According to (63), some compounds, including eugenol (clove aroma), are presented as the most odor-active components. In return, α-copaene exhibited a cytotoxic effect on MCF-7 breast carcinoma cell lines (64). In agreement with the previously mentioned study, the aromatic profile of *P. ruderale* varied greatly, depending on its geographic origin. A study carried out by Fonsceca et al. (65) on the composition of Bolivian *P. ruderale* oil showed that the leaf oil had monoterpene sabinene (64%) as the major constituent; the leaf oil from Mexican *P. ruderale* had limonene (71.4%) as the major constituent, as did the oil of plants from Brazil (74.6%).

Both studies mentioned were carried out with the hydrodistillation extracts, which is very different from the handling of volatiles in this study. HS-SPME/GC-MS allows more genuine volatiles and aromas to be obtained. Within the limitations of the existence of standards and a managed internal library, the majority component found was p-cymene, followed by γ-terpinene. However, the family of fatty alcohols was not detected. Being native to the Western Hemisphere, *P. ruderale* from the Valencian coast has changed its volatile profile in the first approach of this study and may be an interesting hypothesis for further studies. These results allow us to observe how this species shows considerable plasticity of chemotype change as a function of geoclimatic conditions.

The aromatic description of volatiles present in considerable amounts is characterized as “minty/piney” or “terpenic/tropical herbal” for γ-terpinene and its flavor type as “terpenic/citrus/oily green” (47, 66), as well as “herbal/floral” or “citrus/sweet” aroma for (Z)-β-ocimene (66, 67). The organoleptic properties of α-terpinene are described as the odor type “woody, herbal, and medicinal,” and its flavor type as “terpenic/spicy/sharp/minty” (47), or it may be “citrus-like/herbaceous/terpeny” (68). The volatile component p-cymene can be described as the most odor-active component due to its high level in this study, and its characteristics are described as having a “fragrant/sweet/fresh/herbaceous note” (68). All these aromatic characteristics correlate with favorable organoleptic attributes, in addition to providing an antimicrobial effect characteristic of monoterpenes and sesquiterpenes (69).

Another species different from the others was *P. oleracea*, which was confirmed by the heatmap of volatile compounds. It is perhaps the plant with the greatest studies carried out on its volatile compounds. The differentiating aroma in our study was 2-isopropyl-3-methoxypyrazine and its homolog 2-sec-butyl-3-methoxypyrazine, reaching the highest amount among the individual compounds of *P. oleracea*. In contrast with a study carried out by Dabbou et al. (70) with Tunisian purslane leaves, where the pyrazine family was the minority and oxygenated monoterpenes the majority, in our study the pyrazine family was predominant, along with monoterpenoids. The same authors emphasize that some volatile compounds act as bioactive components, with pharmacological effects such as anti-inflammatory, hypoglycemic, and muscle relaxant, among others. According to (71), Chinese purslane had the presence of benzeneacetaldehyde and linalool among its volatile compounds; the same ones were found in our study. Considering that this plant is very popular in traditional Chinese medicine, it is possible that some of the volatile compounds have pharmacological activity. Among chemical families, pyrazines and alcohols stood out above all. As (72) study indicates, pyrazine family compounds have strong antibacterial activity and could significantly inhibit or kill common enteropathogenic bacteria. This consideration could require future studies on the pyrazine family of Valencian *P. oleracea*, even more so since one component of this family was the differentiating aroma and it is found in green bell peppers and peas (62). Other individual compounds present in considerable amounts were linalool and 1-butanol, 3-methyl. The first is characteristic in basil (*O. basilicum* L.), coriander (*C. sativum* L.), and thyme (*T. vulgaris* L.), while the second is found in tomato juice (73). The aromatic descriptors for linalool are “flowery/rose”; for 1-butanol, 3-methyl they are “chemical/harsh/stakes” (74) and “fruity/malty” (75).

The largest group of chemical compounds was concentrated in the species *S. media, S. oleraceus*, and *C. album*. This group was characterized by a more homogeneous distribution of the chemical families identified in each of the volatile profiles and the absence of differentiating aromas. *S. oleraceus* was a species where all the volatile chemical families were found, especially organosulfur compounds such as dimethyl sulfide. Along with its counterpart, dimethyl trisulfide, it is identified as the main aroma component in cooked Brassicaceous vegetables and has a strong and unpleasant aroma, although odor thresholds vary considerably for different chemicals. In contrast, dimethyl sulfide of kale (*B. oleracea*), with other compounds, contributed to roasted/sulfur-like/pungent and sweet aroma characteristics (44). Therefore, this component could be considered as a differentiating aroma for *S. oleraceus*. However, some volatile compounds present in trace amounts can contribute significantly to the characteristic flavor and aroma of fresh foods (76). Other abundant families were benzenoids and medium-chain aldehydes. At the individual level, qualitative variability of compounds such as benzeneacetaldehyde was detected, much higher in *S. oleraceus* vs. *S. media* and *C. album*, its odor description being “floral/herbal/honey/cocoa” (77). The components without a significant level of differences between the three species were 2.4-hexenal, with a “fruity/sweet/fresh” odor note (78), and 2.4-heptadienal-(E, E), one of the lipid-degraded compounds with “metallic and rancid off-flavor” aromatic notes (79), or with the description “fatty/nutty/hay/green/oily” (80). As a particularity of each species, the high content of medium-chain aldehydes stands out in *S. media* and *C. album*, followed by the families of fatty alcohols and benzenoids, respectively. Meanwhile, at the individual level, 1-hexanol emerged with a higher amount in *S. media* compared to the other two species of the group. Likewise, the presence of benzyl isothiocyanate stands out in the three species without significant differences. There is also a considerable amount of trans-β-ionone in *S. media* compared to its group counterparts, and there is a presence of trans-2-hexenol, especially in *S. media*, followed by *S. oleraceus*, with both exceeding the level of C. *album*. The odor description for trans-β-ionone, the volatile from the sesquiterpenoids family, is “woody/violet/fruity” (66). Alternatively, the natural occurrence of trans-2-hexenol is reported in many foods such as allium species, cabbage, chamomile, and lettuce, among others (81). Some volatile production is related to disrupted tissue (membrane breakdown), especially the trans-2-hexenol, for which the aroma is described as “green/leafy” (82, 83).

In general, many C_10_ monoterpenoids and C_15_ sesquiterpenes comprise the most abundant group of compounds present in aroma profiles. In some cases, there are also key compounds determining the characteristic aroma (39). This study has corroborated the predominance of monoterpenoids as a majority family, together with benzenoids and medium-chain aldehydes families. However, the maturity of the plants and their integrity also affect the presence of some volatiles in the aroma profiles of plants. The volatile analysis showed a difference in the relative abundance of the multiple chemical families and their components found in this study. It is known that the main volatiles have their contribution to aromas perceived from food. However, minor volatiles also contribute to organoleptic qualities such as odor and taste, an example of which is (E)-β-ocimene, contributing delicate and elegant aroma characteristics (66). The study confirmed that the aromatic characteristics of the plants under the same geoclimatic conditions show a high variety of volatile compounds. The specific aroma could be used as a biochemical marker of quality characteristics. Sensory aroma analysis as an organoleptic quality of these plants could be included in future research to assess their acceptability and clarify whether aroma differentiation is due to a combination of intensity and relative abundance of some individual volatiles.

## Conclusion

This study is the first report to assess the volatile profiles and identification of differentiating aromas of the seven selected plants that provide a basis for characterization of their organoleptic qualities. Assessment of the volatile profiles was carried out using the HS-SPME/GC-MS method, which was demonstrated to be appropriate for the analysis of volatile compounds in the seven edible plants as an initial approximation in the aromatic quality. These undervalued species showed differences in their aromas, which constitutes an appreciable organoleptic characteristic of the wild species, together with other secondary metabolites present (polyphenols and total acidity). In general, the results obtained in the present work corroborate that the fresh leaves of the studied species are identified by their own organoleptic quality due to the great variability of their volatile profiles that include the constituents with possible bioactive characteristics. These metabolites were grouped into more abundant families, which turned out to be monoterpenoids and benzenoids. The results reveal that the studied undervalued species could be a suitable choice as an alternative to conventional vegetables and as possible material in sustainable production systems. In addition, these plants, due to their organoleptic values, such as flavor and aroma, could contribute to the diversification of gastronomic ingredients and could collaborate in the provision of a healthy and beneficial diet for the health of consumers and the benefit of the planet.

## Data Availability Statement

The raw data supporting the conclusions of this article will be made available by the authors, without undue reservation.

## Author Contributions

TF and MR planned the study, curated the data, and drafted the manuscript. MR supervised the research. TF, MG-M, and EM-P performed the volatile detection and quantification experiments. TF performed the statistical analyses. All authors contributed to the article and approved the submitted version.

## Conflict of Interest

The authors declare that the research was conducted in the absence of any commercial or financial relationships that could be construed as a potential conflict of interest.

## Publisher's Note

All claims expressed in this article are solely those of the authors and do not necessarily represent those of their affiliated organizations, or those of the publisher, the editors and the reviewers. Any product that may be evaluated in this article, or claim that may be made by its manufacturer, is not guaranteed or endorsed by the publisher.
